# Prevalence of dyslipidemia and associated risk factors among adults aged ≥35 years in northern China: a cross-sectional study

**DOI:** 10.1186/s12889-020-09172-9

**Published:** 2020-07-06

**Authors:** Yunfeng Xi, Liwei Niu, Ning Cao, Han Bao, Xiaoqian Xu, Hao Zhu, Tao Yan, Nan Zhang, Liying Qiao, Ke Han, Gai Hang, Wenrui Wang, Xingguang Zhang

**Affiliations:** 1 The Inner Mongolia Autonomous Region Comprehensive Center or Disease Control and Prevention, 50 Ordos street, Hohhot, Inner Mongolia Province 010000 P.R. China; 2 Public Health College, Inner Mongolia Medical University, Hohhot, Inner Mongolia Province PR China

**Keywords:** Dyslipidemia, Prevalence, Risk factors, Northern China, Cross-sectional study

## Abstract

**Background:**

Cardiovascular disease (CVD) prevalence has increased continuously over the last 30 years in China. Dyslipidemia is an important modifiable risk factor in CVD. We aimed to collect current data on the prevalence of dyslipidemia in northern China and explore potential influencing factors.

**Methods:**

In this cross-sectional study, we selected a representative sample of 65,128 participants aged ≥35 years in Inner Mongolia during 2015–2017. All participants completed a questionnaire and were examined for risk factors. Dyslipidemia was defined according to 2016 Chinese guidelines for adults. The associated factors for dyslipidemia were estimated by multivariate logistic regression analysis.

**Results:**

The age-standardized prevalence of dyslipidemia was 31.2% overall, with 4.3, 2.4, 14.7, and 17.4% for high total cholesterol (TC), low-density lipoprotein cholesterol (LDL-C), triglycerides (TG), and low high-density lipoprotein cholesterol (HDL-C), respectively. The dyslipidemia prevalence was significantly higher in men than women (37.9% vs. 27.5%, *P* < 0.001), but postmenopausal women had a higher prevalence of dyslipidemia components (except low HDL-C). Compared with Han participants, Mongol participants had a lower prevalence of dyslipidemia (29.1% vs. 31.4%, *P* < 0.001). Male sex, living in urban areas, Han ethnicity, smoking, obesity, central obesity, hypertension, and diabetes were all positively correlated with dyslipidemia; alcohol consumption was linked to lower risk of dyslipidemia.

**Conclusions:**

Our study revealed that dyslipidemia is a health problem in northern China. Greater efforts to prevent and manage dyslipidemia, especially in men under age 55 years, postmenopausal women, and people with unhealthy lifestyles or chronic diseases.

## Background

With development of the economy and acceleration of urbanization, combined with aging of the population, the incidence of cardiovascular disease (CVD) has continued to increase over the last 30 years in China [1]. CVD was the top cause of death among Chinese adults in 2016, ahead of tumors and other diseases [2]. A number of studies have revealed that dyslipidemia is an important modifiable risk factor with a key role in CVD. Therefore, early screening and effective control of lipid levels can reduce the morbidity and mortality of CVD, which has useful social value [3, 4]. The prevalence of dyslipidemia in the general population of China aged 18 and older has increased from 18.6% in 2002 to 40.4% in 2012 [5, 6]. Without timely and effective control, the rate of dyslipidemia will continue to rise, leading to a heavy burden of CVD. Therefore, it is important to identify the potential influencing factors of dyslipidemia, to manage this condition and reduce the burden of CVD.

The occurrence and development of dyslipidemia frequently involves a long-term, continuous process [7, 8]. Various factors may play different roles in the entire development process of dyslipidemia. Previous studies on dyslipidemia and associated factors have been conducted in different provinces and regions [5, 9, 10], but large-scale investigations in northern China are scarce. The Inner Mongolia Autonomous Region is located in the north of China and includes 12 cities. Owing to its varying geographical features from east to west, the lifestyle and health problems of Inner Mongolia are representative of northern China. The prevalence and mortality of CVD are at higher levels in the region [2, 11]. However, as an important risk factors for CVD, there are few recent comprehensive epidemiologic studies on dyslipidemia in Inner Mongolia. Our current study was designed to systematically obtain current data on the prevalence of dyslipidemia and to explore its potential influencing factors, to provide clues for the prevention and control of dyslipidemia in northern China.

## Methods

### Study population

Participants in our cross-sectional study were recruited from Inner Mongolia, using a convenience sampling strategy to select six sites (Hohhot, Wuhai, Chifeng, Erdos, Hulun Buir, and Xingan League) during 2015–2017. Each site was selected according to geographical, economic, and ethnic distribution factors. Participants were enrolled if they were aged 35–75 years and lived in one of the selected regions for at least 6 of the previous 12 months. Of 70,380 enrolled participants, 5252 (7.5%) were excluded because of missing data for serum lipids or body mass index (BMI). The study was approved by the ethics committee of Fuwai Hospital Chinese Academy of Medical Sciences (approval number: 2014–574). Written informed consent was obtained from each enrolled participant.

### Data collection and measurement

The investigation included baseline information, physical examination, and laboratory testing. All investigators underwent professional training before the study. Information on demographic and social characteristics (such as age, sex, ethnicity, residential region, marital status, educational level, and family income), medical history (such as hypertension and diabetes), and lifestyle (such as smoking and alcohol consumption) were collected by interview where conducted at local community health service or door-to-door visits to use a standardized questionnaire.

Two consecutive blood pressure measurements were taken and the mean blood pressure value of the two readings was used. For each participant, blood pressure measurement was performed on the right upper arm after 5 min of rest, with the participant in a seated position, using an electronic sphygmomanometer (Omron, HEM-7430). Fasting blood samples were collected after at least 10 h of overnight fasting. Venous blood specimen was collected in Vacutainer tubes containing ethylenediaminetetraacetic acid (EDTA). Fasting plasma glucose (FPG) was analyzed enzymatically using an autoanalyzer (BeneCheck,PD-G001–2). Serum total cholesterol (TC), low-density lipoprotein cholesterol (LDL-C), high-density lipoprotein cholesterol (HDL-C), triglycerides (TG) were measured by an automatic biochemical analyzer (Cardiocheck PA). All laboratory equipment was calibrated.

Height and weight were measured, with participants wearing lightweight clothing and no shoes, to the nearest 0.1 cm and 0.1 kg, respectively. BMI was computed as weight (kg) divided by the square of height (m^2^). Waist circumference (WC) was measured at the level of the navel using a tape measure, to the nearest 0.1 cm.

### Definitions

According to 2016 Chinese guidelines for the management of dyslipidemia in adults [6], participants were defined as having dyslipidemia if they had one or more of the following conditions: TC ≥6.22 mmol/L (240 mg/dL), LDL-C ≥ 4.14 mmol/L (160 mg/dL), HDL-C ≤ 1.04 mmol/L (40 mg/dL), TG ≥2.26 mmol/L (200 mg/dL), or if they were taking anti-dyslipidemia medication. Hypertension was considered with systolic blood pressure (SBP) ≥140 mmHg or diastolic blood pressure (DBP) ≥90 mmHg or reported use of antihypertensive medication [12]. The diagnosis criterion of diabetes mellitus was FPG ≥7.0 mmol/L or having received treatment for diabetes [13]. Participants with BMI ≥28 kg/m^2^ were diagnosed with obesity, and central obesity was identified as WC ≥90 cm in men and WC ≥85 cm in women [14]. Smoking was defined as participants who smoked at least one cigarette per day during the past 12 months. Drinking was defined as drinking alcohol at least one time per month in the past 12 months.

### Statistical analysis

We described continuous variables using mean ± standard deviation (SD) or median and interquartile range (IQR), and the Student *t*-test or Wilcoxon rank sum test was used to compare differences. Categorical variables were shown as proportions and compared using a chi-squared test. The age-standardized prevalence was calculated according to the China 2010 census. To explore potential influencing factors of dyslipidemia, multivariate logistic regression analyses were used to calculate odds ratios (OR) and 95% confidence intervals (CIs). All analyses were conducted using SAS version 9.3 (SAS Institute, Cary, NC, USA). Two-sided *P* < 0.05 was considered statistically significant.

## Results

### Characteristics of participants

A total of 65,128 participants (26,959 men and 38,169 women) aged 35–75 years were included in our study. Characteristics of the study participants according to dyslipidemia status are shown in Table 1. Among participants, 20,719 were diagnosed with dyslipidemia (31.8%), with a substantial imbalance between men and women (37.9% vs. 27.5%). Compared with the non-dyslipidemia participants, those with dyslipidemia were more likely to be men, unmarried, and a current smoker and drinker; have older age, a high education level, higher income, and obesity; and to live in an urban area (*P* < 0.001). The mean levels of BMI, waist circumference, SBP, DBP, FPG, TC, LDL-C, and TG were significantly higher in participants with dyslipidemia than in those without dyslipidemia whereas the mean level of HDL-C was lower in participants with dyslipidemia (*P* < 0.001).

**Table 1:** Basic characteristics of participants according to dyslipidemia status

Variable	Total (*N* = 65,128)	Dyslipidemia (*N* = 20,719)	Non-dyslipidemia (*N* = 44,409)	*P*-value
Age (years), mean ± SD	54.51 ± 9.36	54.78 ± 9.20	54.38 ± 9.44	< 0.001
Male, *n* (%)	26,959 (41.4)	10,229 (49.4)	16,730 (37.7)	< 0.001
Urban, *n* (%)	20,207 (31.0)	7517 (36.3)	12,690 (28.6)	< 0.001
Ethnic group, *n*(%)				
Han	58,628 (90.0)	18,753 (90.5)	39,875 (89.8)	< 0.001
Mongol	5523 (8.5)	1627 (7.9)	3896 (8.8)	
Other	977 (1.5)	339 (1.6)	638 (1.4)	
Married, *n* (%)	59,346 (91.1)	18,839 (90.9)	40,507 (91.2)	0.230
≥ High school education, *n* (%)	17,960 (27.6)	6587 (31.8)	11,373 (25.6)	< 0.001
Household income(¥/year), *n* (%)				
≤50,000	57,713 (88.6)	18,024 (87.0)	39,689 (89.4)	< 0.001
>50,000	7415 (11.4)	2695 (13.0)	4720 (10.6)	
Smoking, *n* (%)	16,060 (24.7)	6018 (29.0)	10,042 (22.6)	< 0.001
Drinking, *n* (%)	12,157 (18.7)	4353 (21.0)	7804 (17.6)	< 0.001
Obesity, *n* (%)	16,300 (25.0)	7148 (34.5)	9152 (20.6)	< 0.001
BMI (kg/m^2^), mean ± SD	25.77 ± 3.51	26.88 ± 3.30	25.26 ± 3.48	< 0.001
Waist circumference (cm), mean ± SD	85.62 ± 10.05	89.16 ± 9.51	84.17 ± 9.90	< 0.001
SBP (mmHg), mean ± SD	140.45 ± 20.77	143.04 ± 20.48	139.23 ± 20.80	< 0.001
DBP (mmHg), mean ± SD	84.72 ± 11.49	86.65 ± 11.43	83.82 ± 11.41	< 0.001
FBG (mmol/L), M (IQR)	6.00 (5.40,6.70)	6.20 (5.60,7.00)	5.90 (5.40,6.56)	< 0.001
TC (mmol/L), M (IQR)	4.48 (3.89,5.14)	4.61 (3.86,5.61)	4.44 (3.90,5.01)	< 0.001
LDL-C (mmol/L), M (IQR)	2.40 (1.90,2.96)	2.48 (1.92,3.24)	2.37 (1.90,2.86)	< 0.001
HDL-C (mmol/L), M (IQR)	1.37 (1.13,1.66)	1.03 (0.92,1.36)	1.47 (1.26,1.74)	< 0.001
TG (mmol/L), M (IQR)	1.31 (0.97,1.86)	2.15 (1.40,2.78)	1.13 (0.88,1.50)	< 0.001

*BMI* body mass index, *SBP* systolic blood pressure, *DBP* diastolic blood pressure, *FBG* fasting blood glucose, *TC* total cholesterol, *LDL-C* low-density lipoprotein cholesterol, *HDL-C* high-density lipoprotein cholesterol, *TG* triglyceride, *IQR* interquartile range, *SD* standard deviation

### Prevalence of dyslipidemia

**Fig. 1:**
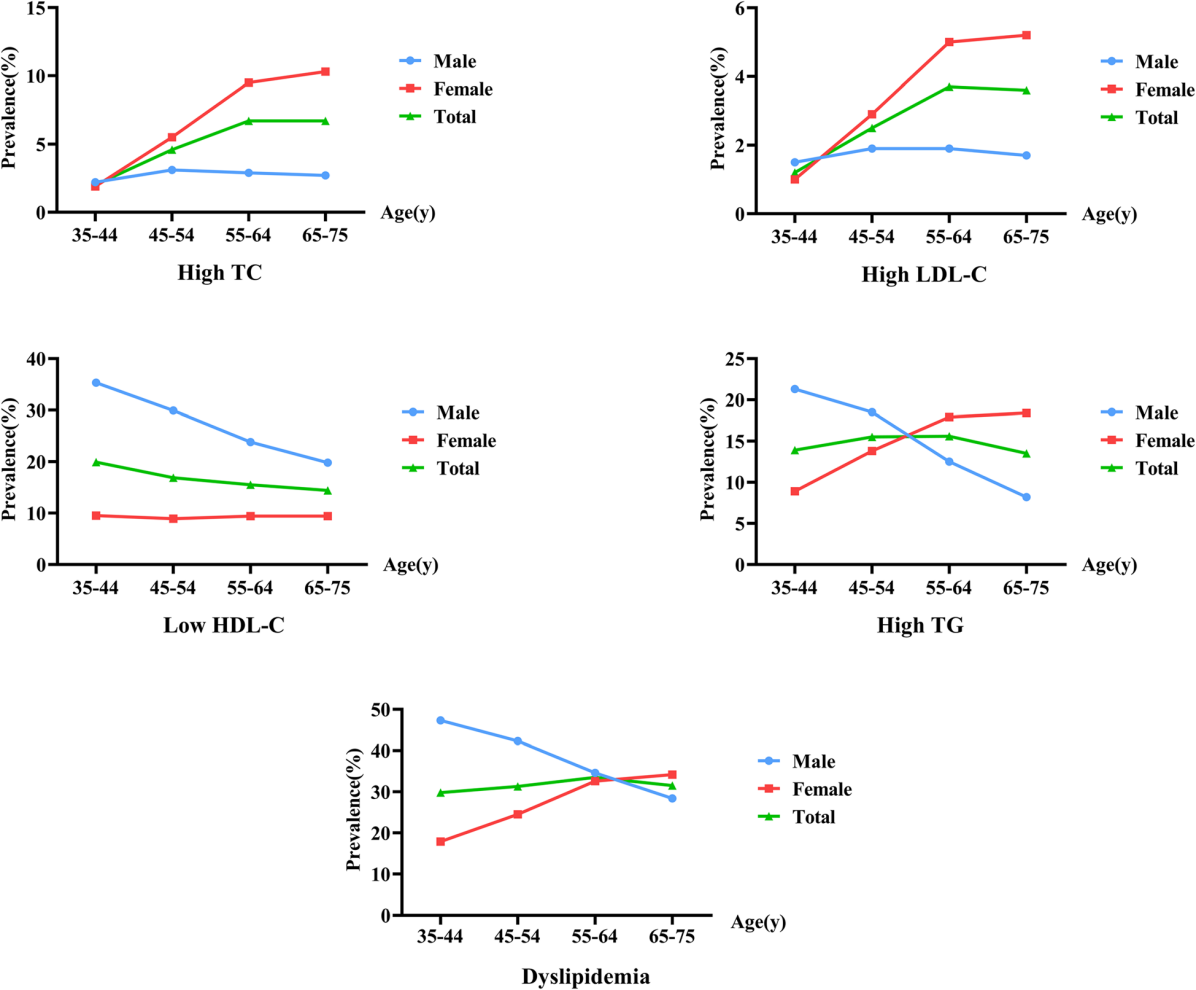
Prevalence of different types of dyslipidemia by sex and age group

Table 2 shows the prevalence of dyslipidemia among subpopulations. The prevalence of dyslipidemia was 31.8%, and the prevalence of elevated TC, LDL-C, TG, and low HDL-C was 5.2, 2.9, 16.5, and 15.0%, respectively. The age-standardized prevalence of dyslipidemia was 31.2%; the age-standardized prevalence of elevated TC, LDL-C, TG, and decreased HDL-C was 4.3, 2.4, 14.7, and 17.4%, respectively. The prevalence of dyslipidemia generally increased with age but was decreased in the age group 65–75 years. The same trend was observed for elevated LDL-C and TG. The prevalence of dyslipidemia was significantly higher in men than in women (*P* < 0.001), but we found a higher prevalence of elevated TC and LDL-C in women than in men. As shown in Fig. 1, elevated TG and dyslipidemia was more prevalent in younger men, and older women had a higher prevalence of dyslipidemia components (except decreased HDL-C), especially postmenopausal women. After age standardization, the prevalence of dyslipidemia varied among ethnic groups. In comparison with Han participants, those with Mongol ethnicity had a lower prevalence of dyslipidemia (29.1% vs. 31.4%, *P* < 0.001), a lower prevalence of decreased HDL-C (16.1% vs. 17.5%, *P* < 0.05), and elevated TG (13.4% vs. 14.8%, *P* < 0.05). Participants who smoked, drank alcohol, and had central obesity, hypertension, or diabetes had a higher prevalence of dyslipidemia and abnormal lipid levels (*P* < 0.001).

**Table 2:** Prevalence of dyslipidemia by subpopulation

	Elevated TC	Elevated LDL-C	Decreased HDL-C	Elevated TG	Dyslipidemia
Total	5.2 (5.0,5.4)	2.9 (2.7,3.0)	16.5 (16.2,16.8)	15.0 (14.7,15.3)	31.8 (31.5,32.2)
Age-adjusted	4.3 (4.1,4.5)	2.4 (2.3,2.5)	17.4 (17.1,17.7)	14.7 (14.4,15.0)	31.2 (30.9,31.6)
Age					
35–44	2.0 (1.8,2.3)	1.2 (1.0,1.4)	19.9 (19.2,20.7)	13.9 (13.3,14.6)	29.8 (28.9,30.7)
45–54	4.6 (4.3,4.9)	2.5 (2.3,2.7)	16.9 (16.4,17.4)	15.5 (15.1,16.0)	31.3 (30.7,31.9)
55–64	6.7 (6.4,7.1)	3.7 (3.4,3.9)	15.5 (15.0,15.9)	15.6 (15.2,16.1)	33.5 (32.9,34.1)
65–75	6.7 (6.2,7.1)	3.6 (3.2,3.9)	14.4 (13.7,15.1)	13.5 (12.9,14.2)	31.5 (30.6,32.4)
*P* for trend	< 0.001	< 0.001	< 0.001	< 0.001	< 0.001
Gender					
male	2.8 (2.6,3.0)	1.8 (1.6,2.0)	26.8 (26.3,27.4)	15.0 (14.6,15.4)	37.9 (37.4,38.5)
female	6.9 (6.7,7.2)	3.6 (3.4,3.8)	9.2 (8.9,9.5)	15.0 (14.6,15.3)	27.5 (27.0,27.9)
*P* value	< 0.001	< 0.001	< 0.001	0.906	< 0.001
Residence					
Rural	5.0 (4.8,5.2)	2.5 (2.4,2.7)	14.1 (13.8,14.4)	14.4 (14.1,14.8)	29.4 (29.0,29.8)
Urban	5.7 (5.4,6.0)	3.6 (3.4,3.9)	21.8 (21.2,22.4)	16.2 (157,16.7)	37.2 (36.5,37.9)
*P* value	< 0.001	< 0.001	< 0.001	< 0.001	< 0.001
Ethnic group					
Han	5.2 (5.0,5.3)	2.8 (2.7,3.0)	16.6 (16.3,16.9)	15.1 (14.8,15.4)	32.0 (31.6,32.4)
Mongol	5.8 (5.1,6.4)	3.1 (2.6,3.6)	15.0 (14.0,15.9) ^*^	13.6 (12.7,14.5) ^*^	29.5 (28.3,30.7) ^*^
Other	5.0 (3.6,6.4)	3.2 (2.1,4.3)	20.6 (18.0,23.1) ^*#^	15.1 (12.9,17.4)	34.7 (31.7,37.7) ^**#**^
*P* value	0.163	0.444	< 0.001	0.008	< 0.001
Smoking					
No	5.8 (5.6,6.0)	3.1 (2.9,3.3)	13.7 (13.4,14.0)	14.8 (14.4,15.1)	30.0 (29.6,30.4)
Yes	3.5 (3.3,3.8)	2.1 (1.9,2.3)	25.2 (24.5,25.8)	15.7 (15.2,16.3)	37.5 (36.7,38.2)
*P* value	< 0.001	< 0.001	< 0.001	0.003	< 0.001
Drinking					
No	5.4 (5.3,5.6)	2.9 (2.8,3.1)	15.4 (15.1,15.7)	14.4 (14.1,14.7)	30.9 (30.5,31.3)
Yes	4.2 (3.9,4.6)	2.5 (2.2,2.8)	21.3 (20.6,22.1)	17.7 (17.0,18.3)	35.8 (35.0,36.7)
*P* value	< 0.001	0.005	< 0.001	< 0.001	< 0.001
Obesity					
No	5.0 (4.8,5.2)	2.8 (2.6,2.9)	13.9 (13.6,14.2)	12.5 (12.2,12.8)	27.8 (27.4,28.2)
Yes	5.9 (5.5,6.3)	3.1 (2.8,3.3)	24.2 (23.6,24.9)	22.6 (21.9,23.2)	43.9 (43.1,44.6)
*P* value	< 0.001	0.051	< 0.001	< 0.001	< 0.001
Central obesity					
No	4.4 (4.2,4.6)	2.4 (2.3,2.6)	11.9 (11.5,12.2)	9.9 (9.6,10.2)	21.5 (21.0,21.9)
Yes	6.2 (5.9,6.4)	3.3 (3.1,3.5)	21.9 (21.5,22.4)	21.0 (20.5,21.4)	38.0 (37.4,38.5)
*P* value	< 0.001	< 0.001	< 0.001	< 0.001	< 0.001
Hypertension					
No	4.0 (3.7,4.2)	2.3 (2.1,2.5)	15.3 (14.9,15.8)	11.1 (10.8,11.5)	26.2 (25.7,26.7)
Yes	6.2 (6.0,6.5)	3.3 (3.1,3.5)	17.4 (17.1,17.8)	18.1 (17.7,18.5)	36.3 (35.8,36.8)
*P* value	< 0.001	< 0.001	< 0.001	< 0.001	< 0.001
Diabetes					
No	4.7 (4.5,4.9)	2.7 (2.5,2.8)	15.3 (15.0,15.6)	12.6 (12.4,12.9)	28.8 (28.4,29.2)
Yes	7.3 (6.8,7.7)	3.7 (3.4,4.0)	21.7 (21.0,22.4)	24.8 (24.1,25.6)	44.5 (43.6,45.4)
*P* value	< 0.001	< 0.001	< 0.001	< 0.001	< 0.001

^*^ Compared with Han: *P* < 0.01; ^#^ Compared with Mongol: *P* < 0.01

### Influencing factors of dyslipidemia

**Fig. 2:**
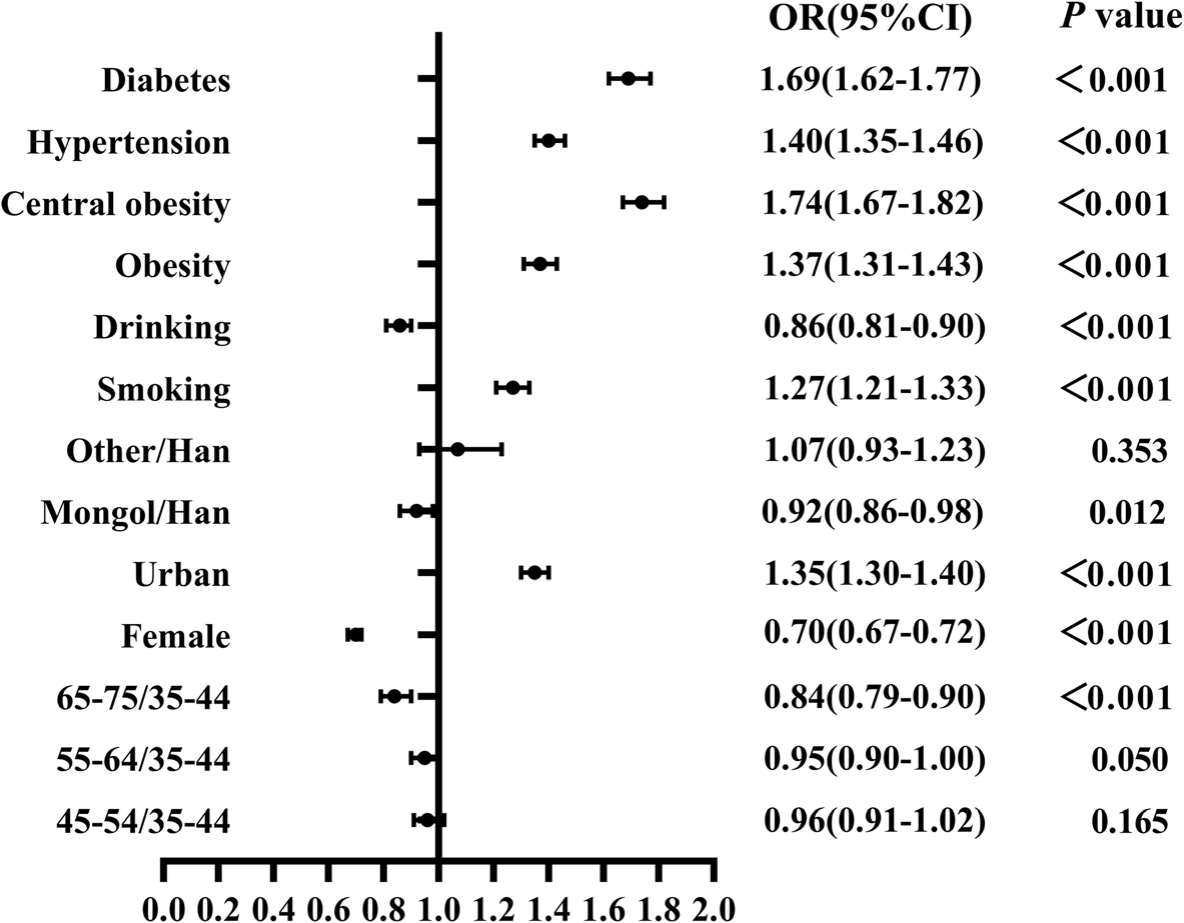
Adjusted Odds Ratios (OR) of influencing factors for dyslipidemia

Figure 2 presents the results of multivariate logistic regression analysis for potential influencing factors associated with dyslipidemia. Participants who were male and those living in urban regions were more likely to have dyslipidemia and smoking was related to a higher risk of dyslipidemia. We also found that participants with obesity, central obesity, hypertension, or diabetes were more inclined to have a positively correlated with dyslipidemia. But our results showed that participants with Mongol ethnicity had a lower risk of dyslipidemia than those with Han ethnicity, and current drinkers were less likely to have dyslipidemia.

## Discussion

A recent study showed that the annual number of deaths owing to CVD increased from 2.51 million to 3.97 million between 1990 and 2016 [2]. CVD has become the leading cause of death in China, particularly in northern China [15]. Dyslipidemia is a well-established risk factor for CVD morbidity and mortality [16]. The prevalence of dyslipidemia increased rapidly from 2002 to 2015 [1]. Identifying the epidemiological characteristics and influencing factors of dyslipidemia is an urgent public health priority. Thus, we carried out the present study of serum lipids, the largest in a representative population of Northern China.

In this study, the age-standardized prevalence of dyslipidemia was 31.2%, and the prevalence rate of abnormal lipid levels was 4.3, 2.4, 14.7, and 17.4% for elevated TC, LDL-C, TG, and decreased HDL-C, respectively. A nationwide investigation showed that the prevalence of dyslipidemia was 34.7% among Chinese adults over age 35 years [17], which was higher than the results of the current study. However, compared with several regional studies [18–20], the prevalence of decreased HDL-C and elevated TG in our study were at higher levels. Located in northern China, Inner Mongolia has different food culture from other regions. Although the consumption of fresh vegetables and fruit has increased in recent years, the diet of local residents was intake meat and animal fat as the main component [21]. A recent cohort study revealed that higher fat intake was related to a greater risk of obesity [22]. Previous studies have demonstrated that the obesity could be related to low HDL-C and elevated TG [23]. While Inner Mongolia was one of the high-prevalence cluster of obesity [24]. This could be explained that Inner Mongolia had the higher prevalence of high TG and low HDL-C. Compared to other Western countries, low HDL-C was the major subtype of dyslipidemia in China [25], and the prevalence of high TG has been shown an increasing trend for Chinese populations in recent years [1]. This was consistent with the situation that the prevalence of dyslipidemia was largely driven by HDL-C and TG in Inner Mongolia. The epidemiological data showed that HDL-C levels were negatively correlated with the risk of atherosclerotic cardiovascular disease (ASCVD) [26], and elevated TG was commonly associated with increased CVD risk [27]. Therefore, monitoring of HDL-C and TG should be strengthened to prevent the incidence of CVD among adults in northern China.

We found that prevalence of dyslipidemia increased with age but was decreased in the age group 65–75 years. Serum lipid abnormalities were more prevalent among men and participants living in urban regions, especially those under age 55–64 years. The prevalence of dyslipidemia, low HDL-C and high TG were decreased with age in men, and the different types of dyslipidemia (except decreased HDL-C) were more prevalent in postmenopausal women. The similar situations that occurred in men among different age groups have been shown in past studies [28, 29]. Although the reason has not been confirmed, in our speculation, men may be more susceptible to risk factors when they are younger, owing to higher intake of fatty foods, greater exposure to unhealthy lifestyles, and a lack of appropriate health knowledge, resulting in men being more prone to dyslipidemia. Previous studies have demonstrated that the prevalence of elevated TC and LDL-C in postmenopausal women is higher than in their male counterparts [30], which is consistent with the findings of our survey. Dyslipidemia is more pronounced in older women, perhaps owing to decreased estrogen levels accompanying menopause, which may have an adverse effect on serum lipid levels in postmenopausal women [31]. As is known, dyslipidemia characterized by elevated TC or LDL-C is considered a major risk factor of ASCVD, and lowering LDL-C levels can significantly reduce the risk of morbidity and mortality owing to ASCVD [6, 32]. To enhance the management of dyslipidemia, greater focus is needed for men under age 55 years and postmenopausal women.

We found disparities in lipid abnormalities according to ethnicity. The prevalence of decreased HDL-C, elevated TG, and dyslipidemia was higher in Han than in Mongol participants, and multivariate analysis showed that people with Mongol ethnicity had a lower risk of dyslipidemia. This finding differs from those of previous studies [33, 34]. There are different genetic backgrounds, cultures, customs, and food consumption patterns between the two ethnic groups [35]. Mongolian tend to eat more meat, milk and dairy products [34]. Some studies conducted in different countries have found that more frequent consumption of milk and dairy products could decreased the risk of dyslipidemia (elevated TG or declined HDL-C levels) [36, 37]. High consumption of milk and dairy products might related to the lower prevalence of high TG and low HDL-C levels in Mongolian.

We identified several factors that were associated with dyslipidemia in our study population. Our results that smoking, obesity, central obesity, hypertension, and diabetes can elevate the risk of dyslipidemia has been confirmed in many previous studies [29, 38]. We also found that current drinkers had a lower risk of dyslipidemia; a similar result was reported by Song [39]. Epidemiological evidence has revealed that moderate alcohol consumption can have beneficial effects on serum lipids, such as elevated HDL-C and decreased LDL-C [40], lowering the TG/HDL-C ratio [41], and that polymorphisms in alcohol-metabolizing enzymes can modify the association between alcohol intake and serum lipids [42], which may reduce the risk of CVD events [43]. However, specific levels of alcohol consumed were lacking in our study; therefore, the influence of drinking on serum lipids must be investigated further. It is recommended that people with chronic diseases or unhealthy lifestyles should be continuously monitored and managed, to effectively control dyslipidemia as much as possible.

There are several potential limitations in our study. First, a cross-sectional design rather than prospective cohort was used; thus, only associations rather than causal relationships could be determined. Second, given a lack of detailed data of dietary, alcohol consumption, physical activity, and family history of dyslipidemia, the associations of these with serum lipids could not be examined in the current study. Third, it is difficult to avoid recall bias and unmeasured confounding owing to collection of information using a questionnaire and measurement. Furthermore, a non-representative sample was used in the study. Therefore, it is not fully generalizable to all adults aged 35 years or above in northern China.

## Conclusions

In summary, our study revealed that dyslipidemia is a health problem in northern China, and the dominant component of dyslipidemia was decreased HDL-C and elevated TG in people aged ≥35 years. We should continuously monitor and manage lipid levels in the elderly. At the same time, the threshold of prevention should be moved forward. Special attention should be pay on the changes in lipid levels for men under age 55 years and for postmenopausal women. Dyslipidemia was more prevalent in people with Han ethnicity than those with Mongol ethnicity. Interventions for factors such as smoking, obesity, central obesity, hypertension, and diabetes can reduce the risk of dyslipidemia, and the association between drinking and serum abnormality requires further exploration. Greater efforts to prevent and manage dyslipidemia in northern China are critical to preventing the growth rate of CVD from becoming difficult to control in the future.

## Data Availability

The raw data of the study is currently not available to publicly share because further research is underway. However, the corresponding author will consider sharing data on reasonable request.

## Abbreviations

CVD: Cardiovascular disease
TC: Total cholesterol
LDL-C: Low-density lipoprotein cholesterol
TG: Triglycerides
HDL-C: High-density lipoprotein cholesterol
BMI: Body mass index
FPG: Fasting plasma glucose
WC: Waist circumference
SBP: Systolic blood pressure
DBP: Diastolic blood pressure
ASCVD: Atherosclerotic cardiovascular disease
